# Anxious Attachment Mediates the Associations Between Early Recollections of Mother's Own Parental Bonding and Mother–Infant Bonding: A 2-Month Path Analysis Model

**DOI:** 10.3389/fpsyt.2021.682161

**Published:** 2021-07-05

**Authors:** Maor Kalfon Hakhmigari, Yoav Peled, Haim Krissi, Sigal Levy, Maayan Molmen-Lichter, Jonathan E. Handelzalts

**Affiliations:** ^1^School of Behavioral Sciences, The Academic College of Tel Aviv-Yafo, Tel Aviv, Israel; ^2^The Helen Schneider Hospital for Women, Rabin Medical Center, Petah Tikva, Israel; ^3^Sackler Faculty of Medicine, Tel Aviv University, Tel Aviv, Israel; ^4^Statistics Education Unit, The Academic College of Tel Aviv-Yafo, Tel Aviv, Israel; ^5^Psychiatry Department, University of Michigan, Ann Arbor, MI, United States

**Keywords:** mother–infant bonding, parental bonding, adult attachment, postpartum, childbirth

## Abstract

Parental bonding (recollection of own parents' parenting), adult attachment, and mother–infant bonding are all closely related yet distinct concepts of the parent–child relationship, sometimes used interchangeably in the literature. This study aimed to examine the associations between these concepts in a longitudinal path analysis design. A total of 262 postpartum women who gave birth at the maternity ward of a large tertiary health center in Israel completed a demographic questionnaire, the Experiences in Close Relationships Scale (ECR), the Parental Bonding Instrument (PBI) at 1–4 days postpartum, and the Postpartum Bonding Questionnaire (PBQ) at 2 months postpartum. Parental care factor (PBI) was found to be associated with mother–infant bonding (PBQ), directly and indirectly through insecure anxious attachment (ECR). Denial of autonomy factor (PBI) was found to be associated with mother–infant bonding (PBQ) only through insecure anxious attachment (ECR). Encouragement of behavioral freedom factor (PBI) was found to be associated with mother–infant bonding (PBQ) in a simple correlation but not in the complete model. The results highlight the intergenerational aspects of parenting and suggest that early childhood interventions with parents may have a long-term impact on child-rearing though generations, and by that on children's development.

## Introduction

Parental bonding, adult attachment, and mother–infant bonding are central concepts of the parent–child relationship (across generations) and are all related to children's development, growth, and wellbeing. These similar yet distinct concepts are sometimes used interchangeably in the literature while not clearly and consistently treated as different concepts (1–4).

In the present study, we aim to expand the understanding of the associations between these concepts by examining how parental bonding and adult attachment are associated with mother–infant bonding. Using a longitudinal path analysis design, we examine whether women's adult attachment orientations measured shortly after birth possibly mediate the associations between parental bonding (the new mother's recollection of the way she was mothered) and mother–infant bonding (her cognitions and feelings toward her infant) at 2 months postpartum. To our knowledge, this study is the first to describe the paths between these close yet different concepts using a longitudinal design.

Mother–infant bonding refers to the emotions and the feelings a mother has toward her infant and herself as a parent. Bonding is believed to emerge during pregnancy or immediately after birth (1). The quality of mother–infant bonding is considered central to infant wellbeing and the child's cognitive and emotional development (5, 6) and was found as an important factor in mothers' later relationships with their children (7–9). Postpartum bonding was found to be associated with a host of factors such as abuse in childhood, family psychiatric history, mother's psychopathology, as well as personality variables [e.g., (6, 10, 11)]. In this study, we focused on the possible associations of mother–infant bonding with the mother's caring model as evolving from her experiences with her own mother, experiences that may have shaped her internal working models of relationships—attachment orientations.

Although similar in name and conceptually closely related to mother–infant bonding, parental bonding refers to the way adults retrospectively report the quality of their parents' parenting, i.e., their childhood relationships and experiences with their parents (12). It was found that mothers' parenting history was associated with the quality of parenting they provide to their infants (13), as mothers who remembered being accepted by their mothers as children were more sensitive and less intrusive with their infants (14). On the other hand, lower recalled care in childhood by the own mother predicted higher dissatisfaction with overall own motherhood (15), while maternal experiences of emotional neglect in childhood were associated with more mother–infant bonding impairments (16).

The Parental Bonding Instrument (PBI) is a scale designed for assessing parenting style retrospectively. Despite its widespread use, there is no consensus regarding its factor structure (17). It was initially conceptualized and assessed as compromised from two factors, care and overprotection (12). However, recent studies reported a different factor structure [e.g., (17)]. In this study, we intend to verify the often reported three-factor structure of PBI: care (reflecting perceptions of the mother as warm and understanding), denial of autonomy (reflecting perceptions of the mother as controlling and overprotective), and encouragement of behavioral freedom (reflecting perceptions of the mother as granting autonomy) (11, 17–19). With regard to parental bonding and mother–infant bonding associations, mothers' perceptions of their child-rearing history (both care and overprotectiveness) were found to be associated with mother–infant bonding (20). However, a later study found that only higher overprotection measured during pregnancy was associated with bonding failure in the postpartum period (21). These findings underscore how the retrospective perception of parenting from own childhood may be relevant to the postpartum mother–infant relationship.

According to the Attachment Theory, early experiences with significant others are internalized and formulate working models that shape individuals' behaviors (22, 23). Those early relations create prototypes for later relationships with close others (24). Attachment with mother/primary caregiver, who can be sensitive and responsive to one's need or not be reliably available and supportive, contributes to the development of internal working models (25). These can mediate the relationship between mothers' early experiences to various aspects of current motherhood (3, 26).

Attachment and maternal–infant bonding refer to different aspects of the parent–child relationship, as bonding emphasizes the mother's tie to her infant, and attachment in general refers to the child's tie to his mother and other caregivers (1, 9). Several studies examined the association between those concepts with inconclusive results pattern. On the one hand, during the transition to motherhood, insecure attachment (anxious and avoidant) was associated directly with bonding difficulties among parents (27) and indirectly mediated by postpartum depression among mothers (28) or mediated by parenting stress (29). On the other hand, Van Bussel et al. (9) reported a weak correlation between mother–infant bonding and attachment style while maternal romantic attachment style predicted attachment with the fetus in the antenatal, but not with the baby in the postpartum period (30) and only women with a dual/disorganized attachment style reported lower bonding than with women with secure and insecure attachment styles (31). This inconclusive results pattern may be explained by the different measures used for attachment and bonding.

In this study, we refer to adult attachment orientations (32)—the internal working models of the mother herself. In order to examine these internal working models in adulthood, researchers focused on a *person's attachment orientation*, which is compromised from two dimensions: *attachment-related avoidance*, which reflects the extent to which a person mistrusts others' intention and therefore defensively strives to maintain behavioral and emotional independence, and *attachment-related anxiety*, which reflects the extent to which a person worries that others will not be available in times of need and anxiously seeks love and care (33).

Past research that examined the relationship between adult attachment and parental bonding during the transition to motherhood revealed that mothers' recollections of their own mothers as supportive and non-intrusive differentiated between securely and insecurely romantic attached participants (3). A recent study reported correlations between recalled care and overprotection in childhood (in opposite directions) and both anxious and avoidant attachment in a sample of pregnant women (15). These findings adhere to the results of several other studies that examined the relationship between parental bonding and adult attachment in diverse populations (2, 34–36). It should be noted that we were interested in the association of recollections of parental bonding and adult attachment orientation, rather than the association of parental bonding and attachment to the fetus or attachment behaviors that are often studied (28, 37).

This study aimed to examine the associations between parental bonding, adult attachment orientations, and maternal–infant bonding of postpartum mothers. Despite the review presented here, to the best of our knowledge, although close by describing parent–child relationships (inter-generationally), these are distinct concepts. Parental bonding refers to the way the mother (in our sample) recalls the relationship with her mother in childhood (12). Her adult attachment orientation refers to the way these recollections, among other factors, have shaped the working models she has for relationships with others in adulthood (33). Finally, mother–infant bonding refers to the emotions and the feelings a mother has toward her infant and herself as a parent (1).

**Figure 1:**
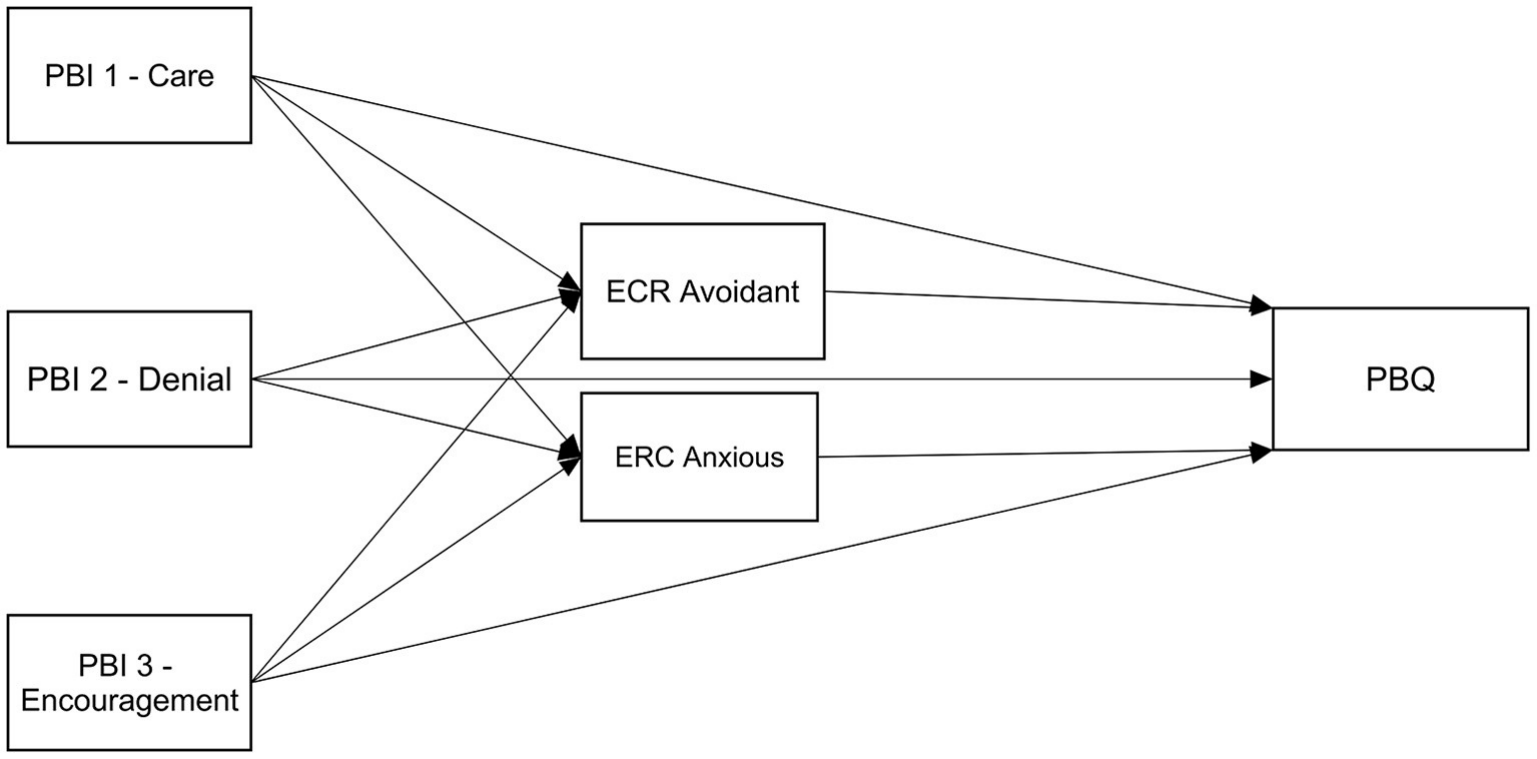
Hypothesized mediation model; Insecure attachment orientations (ECR) will mediate the association between parental bonding (PBI) and mother-infant bonding (PBQ).

We propose a model in which the mother's childhood recollections of her mother parenting her influence her bonding with her infant, and this association is mediated by her adult attachment orientation. While we study parental bonding and adult attachment orientations shortly after birth (along with other demographic and obstetric control variables), we measure their influence on mother–infant bonding at 2 months postpartum. Hence, we hypothesize that insecure attachment orientations (both anxious and avoidant) will mediate the association between recalled parenting by the mother and mother–infant bonding, as higher levels of parental bonding (PBI) will be associated with lower levels of anxiety or avoidance attachment orientation, which will be associated with better mother–infant bonding at 2 months postpartum (see Figure 1).

## Materials and Methods

### Sample

**Figure 2:**
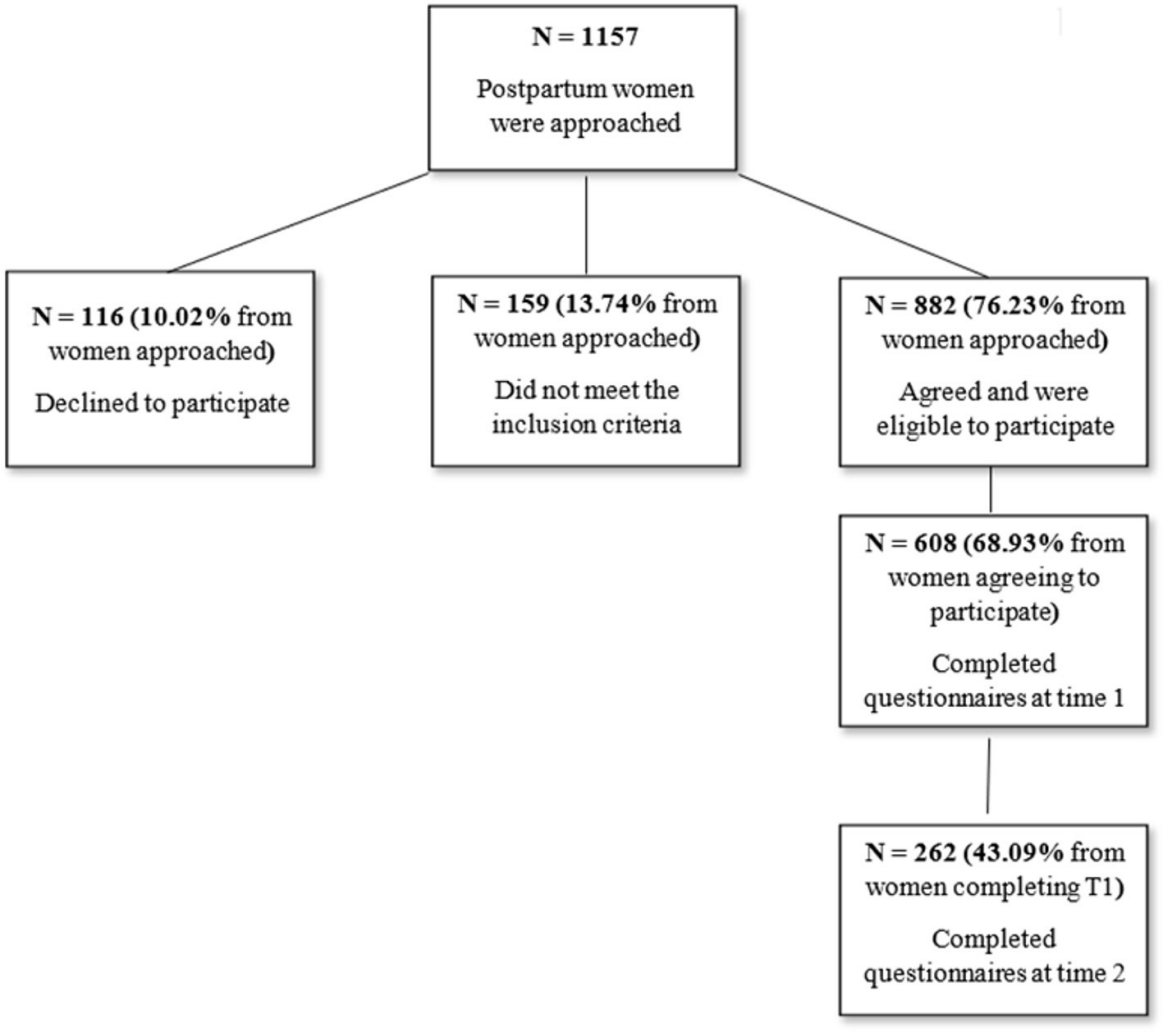
Recruitment and data collection.

The final sample included 262 postpartum women who gave birth in the maternity wards of the Rabin Medical Center (RMC), a large tertiary health center in Israel. Eligibility criteria included delivering at least at 37 weeks' gestation, a singleton pregnancy, and Hebrew speaking. Information about recruitment, data collection, and dropout rates can be seen in Figure 2. Comparing dropouts (women who completed only the first time point) to women who completed the second time point with sufficient data showed that completers were less avoidant (ECR) [*M* = 2.5, *SD* = 0.9 vs. *M* = 2.8, *SD* = 1.0, *F*_(1, 481)_ = 8.6, *p* = 0.003], lower in PBI denial factor [*M* = 0.7, *SD* = 0.1 vs. *M* = 0.9, *SD* = 0.9, _F(1, 540)_ = 12.0, *p* = 0.001], and older [*M* = 32.0, *SD* = 4.7 vs. *M* = 31.0, *SD* = 5.4, F_(1,587)_ = 4.5, *p* = 0.035]. In addition, primiparous women were less likely to complete the second time point (41 vs. 50%, *p* < 0.001), as were women with less than university educational level (38 vs. 53%, *p* < 0.001) and with below average income (43 vs. 56%, *p* = 0.002).

The average age of the participants was 31.7 (±4.8), most (94%) were married, 84% were born in Israel, and 92% were Jewish. Most women (76%) had vaginal births, 9% had elective cesarean section, 10% had emergency cesarean sections, and 6% had an assisted vaginal birth. Just over half of all women (53% of the whole sample, 61% if excluding women who had elective CS) were administered an epidural, and 43% (49% if excluding women who had elective CS) had oxytocin for labor augmentation. For participants' demographic data, see Table 1.

**Table 1:** Sample demographic characteristics and correlations with PBQ score.

	***N* (%)**	***M* (*SD*), Range**	**Correlation with PBQ**
**Age**		31.7 (4.8), 20-43	0.03
**Primiparous**			0.22^**^
Yes	67 (26)		
No	195 (74)		
**Higher education**			0.14^*^
Yes	187 (71)		
No	75 (29)		
**Income level**			−0.05
Average or below	145 (55)		
Above average	112 (43)		
Unknown	5 (2)		
**Marital status**			−0.09
Married	246 (94)		
Not married	16 (6)		
**Psychiatric diagnosis**			0.26^**^
Yes	10 (4)		
No	252 (96)		
**Unplanned birth type**			0.18^**^
Yes	42 (16)		
No	220 (84)		

* *p < 0.05*,

** *p < 0.01*.

### Procedure

The study is part of a larger longitudinal study aimed at understanding associations between factors associated with birth and postpartum mental health during the first 6 months postpartum conducted between July 2018 and July 2019. Ethical approval for this study was obtained from the RMC and the Academic College of Tel-Aviv Yaffo institutional review boards. Research assistants, graduate students with appropriate training in research ethics, approached all women at the maternity ward on a random day of the week, and after giving informed consent, the participants answered questionnaires at two time points:

T1 (1–4 days postpartum) in person at the maternity ward—obstetric data were taken from the medical files and women completed demographic questions, the Experiences in Close Relationships Scale (ECR) and the PBI.

T2 (2 months postpartum)—using online questionnaires, the participants completed the Postpartum Bonding Questionnaire (PBQ).

Participants who did not respond to the email invitation were reminded once with a phone call. Questionnaires and data output were generated using Qualtrics© 2015 (Qualtrics, Provo, UT, USA; http://www.qualtrics.com).

### Measures

#### Sociodemographic Questionnaire

Sociodemographic questionnaire included questions about age, education level, marital/co-habiting relationship, income level (as compared to the national average per household at the time of the study), religious affiliation, country of origin, and the history or current existence of psychiatric disorders.

#### Obstetric Data

Obstetric data were extracted from medical records, recording number of previous births, infertility treatments, pregnancy risks, past abortions or miscarriages, and current birth data: type of birth as well as epidural and oxytocin administration. We treated “birth type” as a dichotomous variable for statistical reasons and according to relevant literature that claims that the importance of the birth type variable is whether it was expected or not (38–40). Thus, vaginal birth and elective cesarean sections are considered “Expected birth,” while emergency cesarean section and vaginal assisted birth are considered “Unexpected birth.” Being primiparous, having higher education, having above average income, being married, having a psychiatric diagnosis, and having an unplanned birth type were dummy-coded as “1” while other values were coded as “0.”

#### Parental Bonding

Parental bonding was assessed with the PBI (12), a 25-item scale designed to measure retrospective recollections and perceptions of early parental attitudes and behaviors that has been widely used for assessing recollections of parent–child relations (41). We used the Hebrew version (42) and inquired only about recollections of mother's parenting. Participants were asked to rate the degree to which each item describes their mother's early behavior and attitudes, on a four-point scale, ranging from 0 (not at all) to 3 (very much). In order to verify the three-factor solution of the scale, we performed parallel analysis, a more accurate method for determining the number of factors in a set of items than the commonly used criterion of eigenvalue >1, which tends to extract too many factors (43). Parallel analysis indicated three underlying factors. Exploratory factor analysis using principal components analysis with varimax rotation provided a corresponding solution, almost identical to the factors found in a recent large population-based psychometric validation of the scale as well as other studies [see (17)] apart from item 3 that loaded in our study on the encouragement factor and in the mentioned research loaded on both encouragement and care and item 8 that loaded on the denial of autonomy scale in our study and on the encouragement factor in the mentioned study. These three factors were also found in our previous study of different sample (11). The three factors together explained 52% of the variance in the responses (see Table 2): (1) *PBI-Care*, reflecting perceptions of the mother as warm and understanding (12 items; α = 0.90); (2) *PBI-Denial of autonomy*, reflecting perceptions of the mother as controlling and overprotective (7 items; α = 0.79); and (3) *PBI-Encouragement of behavioral freedom*, reflecting perceptions of the mother as granting autonomy (6 items; α = 0.85). Scores were computed by calculating an average for each subscale with higher scores reflecting stronger perceptions. The intercorrelations between the three factors were significant yet moderate (0.23–0.38), supporting their use as three separate factors (see Table 2).

**Table 2:** Factor loadings for principal components analysis of PBI with Varimax rotation.

	**Care**	**Denial**	**Encouragement**
**FACTOR 1**			
Item 6 Was affectionate to me	**0.77**	0.07	−0.29
Item 11 Enjoyed talking things over with me	**0.74**	0.05	−0.22
Item 18 Did not talk with me very much	**0.73**	−0.19	−0.01
Item 12 Frequently smiled at me	**0.70**	0.03	−0.17
Item 1 Spoke to me in a warm and friendly voice	**0.69**	−0.06	−0.28
Item 17 Could make me feel better when I was upset	**0.69**	−0.05	−0.30
Item 5 Appeared to understand my problems and worries	**0.69**	0.04	−0.33
Item 4 Seemed emotionally cold to me	**0.64**	−0.19	−0.01
Item 24 Did not praise me	**0.62**	−0.31	0.00
Item 2 Did not help me as much as I needed	**0.58**	−0.13	0.16
Item 16 Made me feel I wasn't wanted	**0.55**	−0.32	−0.04
Item 14 Did not seem to understand what I needed or wanted	**0.52**	−0.47	−0.24
**FACTOR 2**			
Item 19 Tried to make me feel dependent on her	−0.14	**0.74**	0.07
Item 13 Tended to baby me	−0.09	**0.70**	0.06
Item 20 Felt I could not look after myself unless she was around	−0.17	**0.69**	0.04
Item 9 Tried to control everything I did	−0.24	**0.64**	0.28
Item 8 Did not want me to grow up	0.00	**0.59**	0.01
Item 10 Invaded my privacy	−0.20	**0.57**	0.36
Item 23 Was overprotective of me	0.16	**0.54**	0.19
**FACTOR 3**			
Item 22 Let me go out as often as I wanted	−0.09	0.06	**0.83**
Item 21 Gave me as much freedom as I wanted	−0.09	0.14	**0.82**
Item 15 Let me decide things for myself	−0.22	0.27	**0.72**
Item 25 Let me dress in any way I pleased	−0.08	0.08	**0.69**
Item 7 Liked me to make my own decisions	−0.11	0.25	**0.66**
Item 3 Let me do those things I liked doing	−0.36	0.04	**0.54**
**Cumulative Explained Variance**	**23%**	**52%**	**38%**

*Item loadings in bold indicate the subscale in which the item was included*.

#### Adult Attachment

Adult attachment was assessed by the ECR (44), which assesses the dimensions of anxious and avoidant adult attachment. For the purpose of the study, we used an abbreviated, validated Hebrew version that consists of 24 items divided into two dimensions: anxious (12 items, e.g., “I worry about being abandoned”) and avoidant (12 items, e.g., “I feel discomfort when others get close to me”) (45). Participants rated the extent to which an item described themselves on a seven-point scale, ranging from 1 (strongly disagree) to 7 (strongly agree). A high score indicates higher anxious or avoidant attachment. This scale has been used in previous studies with postpartum women [e.g., (46, 47)]. In the current study, the internal reliability was good (α = 0.84 for anxiety and α = 0.83 for avoidance).

#### Mother–Infant Bonding

Mother–infant bonding was assessed by the Hebrew version of the PBQ (48, 49). This 25-item scale assessed the mother's feelings or attitudes toward her baby (e.g., “I feel close to my baby”). Statements are presented on a six-point scale, ranging from 0 (always) to 5 (never), with reverse coding of positive items. Responses are summed so that higher scores denote **greater** bonding difficulties (poorer bonding). Two items relating to the risk of abuse were not included due to ethical considerations (50). Internal reliability of the total scale was good (α = 0.89).

### Statistical Analysis

Data were described as *M*(*SD*) and range or as counts and percentages. Correlations between the study variables were assessed using the Pearson correlation coefficient. Exploratory factor analysis for determining the factor structure of the PBI questionnaire used principal components analysis with varimax rotation. Items were assigned to factors on which their loading was 0.5 or higher. Path analysis with 1,000 bootstrap samples was used to test the mediation model. Data were analyzed using SPSS v.25 and AMOS v.25. Using the 15 observations per measured variable rule, we concluded that a sample of about 200 observations should be sufficient for testing our hypothesized model including possible covariates.

## Results

Table 1 shows the sample demographics as well as correlations between the demographic characteristics and the outcome variable. The correlations between the study variables, as well as their means and standard deviations, are shown in Table 3. All study variables significantly correlated with each other.

**Table 3:** Descriptive statistics and Pearson correlations between the study variables.

	**2**	**3**	**4**	**5**	**6**	***M* (*SD*)**
1. PBQ	−0.24^**^	0.25^**^	0.17^**^	0.24^**^	0.32^**^	0.4 (0.4)
2. PBI factor 1: Care		−0.23^**^	−0.37^**^	−0.31^**^	−0.27^**^	2.6 (0.5)
3. PBI factor 2: Denial			0.35^**^	0.22^**^	0.33^**^	0.9 (0.5)
4. PBI factor 3: Encouragement				0.15^*^	0.23^**^	0.7 (0.6)
5. Avoidant attachment					0.51^**^	2.5 (0.9)
6. Anxious attachment						2.6 (1.1)

* *p < 0.05*,

** *p < 0.01*.

**Figure 3:**
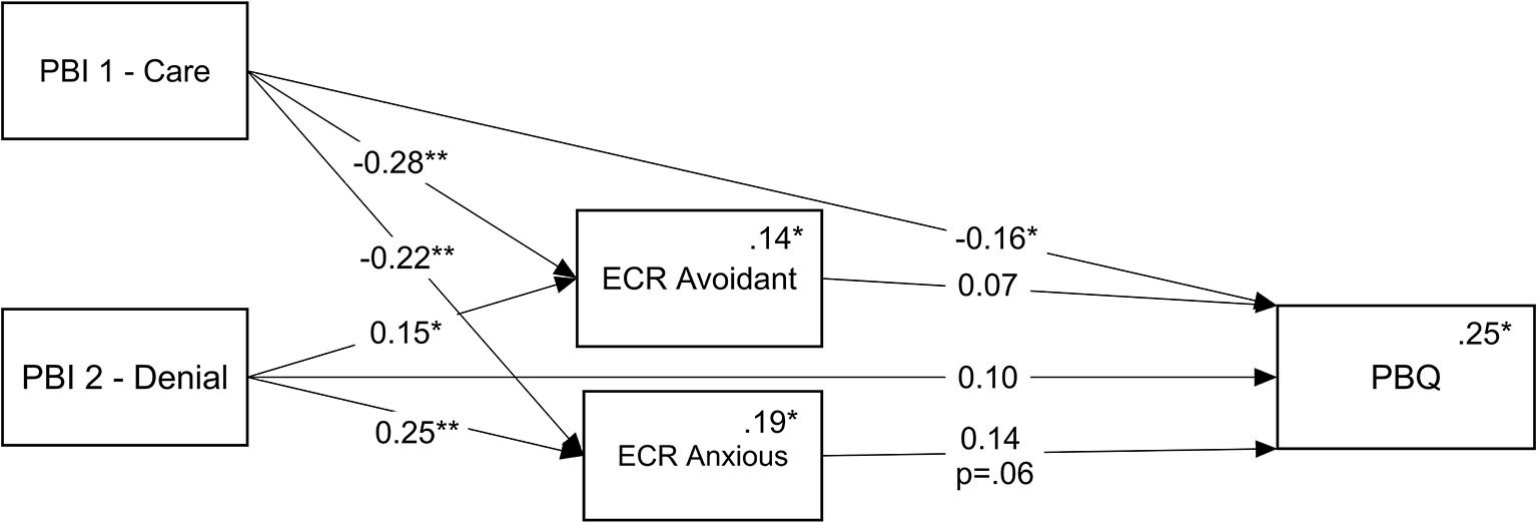
Final mediation model; ECR (anxious attachment orientation) mediating the association between PBI factors (parental bonding care and denial of autonomy) and PBQ. *p < *0.05*, **p < *0.01*. While not shown on the graph, this model was analyzed with being primiparous, education, and having a psychiatric diagnosis as covariates. Numbers above the lines are standardized regression weights, and numbers above the endogenous variables and multiple squared correlations.

Given the correlations shown in Table 1, the hypothesized model was tested with being primiparous, education, psychiatric diagnosis, and birth type as covariates, linked to the study variables with which they had significant correlations. The results showed that birth type and the PBI Encouragement factor had no significant effects on any of the other model variables, and so were excluded from the model. The resulting model is shown in Figure 3. Results show that the model had good fit [$\chi_{\left(3\right)}^{2}$ = 1.95, *p* = 0.58, NFI = 0.99, TLI = 1.04, CFI = 1.00, RMSEA = 0.00] and accounted for 25% of the PBQ variance. PBI Care factor had both direct [β = −0.16, *p* = 0.02, 95% CI = (−0.28, −0.02)] and indirect effects on PBQ, going through anxious attachment [β = −0.03, *p* = 0.03, 95% CI = (−0.08, 0.00)]. We found no direct effect of the PBI Denial factor on PBQ, yet we found an indirect effect through anxious attachment [β = 0.04, *p* = 0.04, 95% CI = (0.00, 0.10)]. While PBI Encouragement factor was significantly correlated with PBQ as can be seen in Table 3, this correlation diminished in the presence of the other factors in the final model. Avoidant attachment did not serve as a mediator despite its correlations with the PBI factors and PBQ (see Table 3), as anxious attachment accounted for the mediation effect.

In sum, we learn that anxious attachment mediates the relationships between PBI factors (Care Denial) and PBQ: lower PBI care factor was related to higher ECR attachment anxiety, which in turn was related to higher PBQ score, resulting in a negative indirect effect. In contrast, high PBI denial factor was related to higher ECR attachment anxiety, which was followed by higher PBQ score, hence the positive indirect effect. In addition, in the final mode, PBI Encouragement factor was not associated with PBQ and avoidant attachment was not found to be a mediator between PBI factor and PBQ.

## Discussion

The current study aimed to examine the associations between parental bonding (PBI), adult attachment orientations, and mother–infant bonding (PBQ) among mothers, in a longitudinal design from childbirth to 2 months postpartum. Our findings indicate that parental bonding care factor was associated with mother–infant bonding both directly and indirectly through anxious attachment, while parental bonding denial of autonomy factor was associated with mother–infant bonding only indirectly through anxious attachment. The third factor, parental encouragement of behavioral freedom was associated with bonding in a simple correlation but was not associated with mother–infant bonding in the complete model. Additionally, parental care and parental denial of autonomy factors were associated with avoidant attachment, though avoidant attachment was not associated with mother–infant bonding. The study findings contribute to the existing literature in several aspects. First, our findings emphasize that parenting models that new mothers have absorbed from their mothers may have shaped their internal working models and, through those, but also directly, may be associated with the way they perceive their bond with their new infant. In particular, we found that childhood recollections of mothering that lack warmth and understanding or are characterized as controlling and overprotective may be associated with higher levels of anxious attachment orientation, which in turn may increase mother–infant bonding difficulties. Our results are in line with previous findings that emphasize that parents' recollections of the way they were parented are important to their postpartum parenthood mental health and psychological wellbeing (51–53). This intergenerational perspective emphasizes that early childhood caretaking experiences of mothers may continue to compromise the mother's capacity to cope during her own parenthood (20) through her internal working models and the bond she perceives with her new infant (28). This finding is of importance as the quality of the maternal infant relationship postpartum, and in particular mother–infant bonding, is considered central to infant wellbeing, cognitive and emotional development, and adaptation throughout life (54–56).

In addition, the findings contribute to the literature regarding the PBI factor structure by replicating the PBI's three factors structure as reported in recent studies [e.g., (17)]. Our study results put a spotlight on parental care, which was the only factor associated directly and indirectly through anxious attachment with mother–infant bonding. It emphasizes parental care as a possible distinct concept from the other two PBI factors (vis-à-vis bonding) and in line with previous studies that report parental care as a clear, cohesive, and stable factor (18, 41, 57). We suggest that anxious attachment orientation may explain the association between parental care and bonding, but our model also alludes to the possible direct association between parental care and mother–infant bonding. It underscores parental care as a significant variable in parenting (15) and as meaningful during the transition to parenthood (11).

In the complete model, the second PBI factor, parental denial of autonomy, was related to mother–infant bonding only through anxious attachment. This finding adds to the literature regarding this factor role, as a previous study found an association between parental denial of autonomy and maternal–fetal attachment (11). The third factor, parental encouragement of behavioral freedom, was not associated with mother–infant bonding in the model. Thus, the third factor, which is relatively new, is yet to be studied with relation to other variables in general and parenting in particular.

Although we hypothesized that both insecure attachment orientations (anxious and avoidant) would mediate the associations between parental bonding and mother–infant bonding, the results indicated differential mediation; only anxious attachment mediated this association in the overall model. It is important to note that we found a simple correlation between anxious as well as avoidant attachment orientations and mother–infant bonding while both parental bonding care and denial factors were associated with both anxious and avoidant attachment orientations. In general, our findings adhere to the literature linking insecure attachment orientations and parenting variables (58) and emphasized that insecure attachment has an important role during the transition to parenthood (59). Our findings demonstrate adult attachment and maternal–infant bonding as different aspects of the intergenerational perspective of parent–child relationship, as adult attachment orientations refer to the internal working models of relationships in adulthood and mother–infant bonding refers to the mother's tie to her infant (1, 9). However, various studies investigating the specific associations of avoidant and anxious attachment orientations and mother–infant bonding report inconsistent findings, similarly to our findings. For example, only avoidance was a significant predictor of mother–infant bonding when controlling for demographic variables and maternal mental health history (29). In another study, there was no direct relationship between attachment and mother–infant bonding; however, anxious attachment was associated with postpartum depression, and depressive symptoms predicted impaired bonding (30). Other studies investigated associations between other attachment orientations (secure attachment and dual/disorganized attachment) with bonding [e.g., (27, 31)]; therefore, more research is clearly needed to elucidate the role of the different insecure attachment orientations vis-à-vis parenting in general and the mediation of the association between parental bonding and mother–infant bonding in particular. Our model suggests that the way mothers report about the mothering they have received in childhood retrospectively may shape their anxious and avoidant orientations, but only anxious orientation was found to have a significant association with bonding to the infant in our model.

Although our study was in a longitudinal design using a relatively large sample, it is not without limitations. First, the study participants were mothers asked to report their childhood recollections of the way their mothers cared for them. Further research is needed both regarding fathers and memories of parenting experiences with fathers. Furthermore, the PBI is a self-report measure of parental bonding reported retrospectively that might be influenced by recall biases, though parental bonding recollections as measured by the PBI exhibited stability over a 20-year period, suggesting that recall biases of parental bonding may be modest (60). Second, in this study we used only self-report measures. Future research could use observational measures of attachment or mother–infant interactions as well as study the participants' mothers and infants to further learn about intergenerational perspectives in more ecological designs. Third, we measured our variables in the first 2 months' time frame, while future research could study parenting perceptions in longer periods after childbirth. Fourth, there was a difference between the first time point assessment that was done in-person and the second time point that was done online, causing a potential confounding. This difference in assessments is a result of following up on a fairly large sample. Recent research of offline vs. online assessment of the PBQ found no difference between assessment modalities in terms of associations with sociodemographic, reproductive, obstetric, and psychological outcomes (61). Lastly, our participants were mostly Jewish, sampled from one health center only, and this may impair our ability to generalize our findings, as parenting practices may be different across cultures.

In conclusion, the present study examined the associations between the close yet distinct concepts of the parent–child relationship: parental bonding, adult attachment orientations, and mother–infant bonding. Our findings demonstrate the associations between those three separate concepts in a longitudinal design, as our model emphasizes that parenting models mothers have received from their mothers may shape their internal working models and those in turn were associated with the way they perceive the bond with the new infant. These findings highlight the intergenerational conceptualization of parenting (62) and emphasize that early childhood interventions with parents might be significant for a long-term impact on the development of future generations.

## Data Availability Statement

The raw data supporting the conclusions of this article will be made available by the authors, without undue reservation.

## Ethics Statement

The studies involving human participants were reviewed and approved by Rabin Medical Center and the Academic College of Tel-Aviv Yaffo institutional review boards. The patients/participants provided their written informed consent to participate in this study.

## Author Contributions

JH, MK, HK, and YP contributed to the conception and design of the study. MM-L and SL organized the database. SL performed the statistical analysis. MK and JH wrote the first draft of the manuscript. JH, MK, and YP wrote sections of the manuscript. All authors contributed to manuscript revision, read, and approved the submitted version.

## Conflict of Interest

The authors declare that the research was conducted in the absence of any commercial or financial relationships that could be construed as a potential conflict of interest.
